# Respiratory Tract Deposition and Distribution Pattern of Microparticles in Mice Using Different Pulmonary Delivery Techniques

**DOI:** 10.3390/vaccines6030041

**Published:** 2018-07-10

**Authors:** Nitesh K. Kunda, Dominique N. Price, Pavan Muttil

**Affiliations:** Department of Pharmaceutical Sciences, College of Pharmacy, University of New Mexico Health Sciences Center, Albuquerque, NM 87102, USA; nkunda@salud.unm.edu (N.K.K.); dnprice@salud.unm.edu (D.N.P.)

**Keywords:** pulmonary delivery, microparticles, vaccines, respiratory tract deposition, IVIS, MicroSprayer^®^ Aerosolizer, BioLite intubation, oropharyngeal aspiration

## Abstract

Pulmonary delivery of drugs and vaccines is an established route of administration, with particulate-based carriers becoming an attractive strategy to enhance the benefits of pulmonary therapeutic delivery. Despite the increasing number of publications using the pulmonary route of delivery, the lack of effective and uniform administration techniques in preclinical models generally results in poor translational success. In this study, we used the IVIS Spectrum small-animal in vivo imaging system to compare the respiratory tract deposition and distribution pattern of a microsphere suspension (5 µm) in mice after 1, 4, and 24 h when delivered by oropharyngeal aspiration, the Microsprayer^®^ Aerosolizer, and the BioLite Intubation System, three-widely reported preclinical inhalation techniques. We saw no significant differences in microsphere deposition in whole body images and excised lungs (at 1, 4, and 24 h); however, the three-dimensional (3D) images showed more localized deposition in the lungs with the MicroSprayer^®^ and BioLite delivery techniques. Further, oropharyngeal aspiration (at 1 h) showed microsphere deposition in the oral cavity, in contrast to the MicroSprayer^®^ and BioLite systems. The studies shown here will allow researchers to choose the appropriate pulmonary delivery method in animal models based on their study requirements.

## 1. Introduction

Inhalational delivery of therapeutics has been utilized for many centuries around the world. Pulmonary delivery is a non-invasive route of administration with many benefits, such as a large lung surface area for absorption (100 m^2^), elevated blood flow, rapid absorption, and avoidance of hepatic first-pass metabolism [1,2]. These benefits of pulmonary drug and vaccine delivery outweigh the challenges which include, most notably, mucociliary clearance, physiological barriers limiting deep lung deposition, formulation difficulties, and variability in inhaler use [2]. With respect to drugs, pulmonary delivery enhances the drug concentration locally and can potentially diminish systemic adverse effects [3]. Similarly, pulmonary vaccination has been shown to generate regional and long-lasting protective immunity within the lung, a common site of infection for many pathogens [4,5,6,7].

One promising strategy to further improve pulmonary therapeutics is to formulate drugs and vaccines in particulate carriers such as micro- and nano-particles to offer benefits such as higher therapeutic efficacy with lower doses, enhanced immune responses due to particle uptake by antigen presenting cells (APCs), improved drug and antigen loading, and adjuvant properties of particulate carriers [8]. Moreover, drug and vaccine antigens are protected from degradation when encapsulated in microparticles and can further be decorated with surface moieties for targeted therapy [9]. Additionally, particulate formulations in the size range of 0.5–5 µm are carried into the lymphatic system by APCs and thus induce strong antibody responses [10,11]. Over the last decade, there have been numerous publications evaluating particulate-based vaccines for pulmonary delivery in preclinical animal models [12,13,14,15,16]. Unfortunately, many of the pulmonary drug and vaccine candidates that are successfully evaluated in preclinical studies do not proceed to clinical trials; the failure to translate preclinical studies into humans is despite a plethora of preclinical studies employing the pulmonary route for therapeutics and vaccine delivery [15,16,17,18,19]. This problem was recently discussed by Muttil and colleagues [20], who suggested that one of the reasons for poor translation of preclinical studies is due to the different mechanisms by which preclinical inhalation devices operate compared to human inhalers. This prompted us to compare the three commonly used preclinical inhalation devices/techniques to provide a better understanding of the literature that employs these devices.

Current preclinical devices for pulmonary administration either employ passive or direct inhalation techniques. Direct inhalation techniques include intratracheal (IT) and intranasal instillation, and tracheostomy [20]. IT instillation offers the advantage of precise dosing of the test agent as compared to passive inhalation techniques, as the therapeutic is placed directly into the upper airway [21]. Additionally, selection of an appropriate animal model is vital to examine the fate of the inhaled drug. Price et al. recently discussed in detail the importance of animal model selection when evaluating an inhaled product in the preclinical stage [20]. Most of the published papers evaluating inhaled drugs employ small animal models such as mice and rats due to ease of availability, affordability, and handling [3,7,22,23,24]. In this study, we evaluated three widely reported preclinical inhalation (direct) techniques: the MicroSprayer^®^ Aerosolizer (Penn-Century, Wyndmoor, PA, USA; recently discontinued), the BioLite Intubation System (Braintree Scientific, Braintree, MA, USA), and oropharyngeal aspiration. We used these delivery methodologies to determine the deposition and distribution pattern of a microsphere suspension (5 µm in size) in the respiratory tract of mice at 1, 4, and 24 h after pulmonary administration.

## 2. Materials and Methods 

### 2.1. Materials

Near-infrared fluorescent Degradex^®^ poly(d,l-lactide-co-glycolide) (PLGA; lactic acid: glycolic acid ratio of 50:50 and a molecular weight of 30 kDa) microspheres were purchased from Phosphorex, Inc. (Hopkinton, MA, USA). Microspheres were 5 µm in size, with a density of ~1.3 g/mL. The fluorescent microspheres had an excitation wavelength (λ_ex_) of 780 nm and an emission wavelength (λ_em_) of 820 nm. The microspheres were supplied as lyophilized powders and were reconstituted in phosphate-buffered saline (PBS, pH 7.4) to achieve a final concentration of 10 mg/mL and sonicated prior to pulmonary administration. 

### 2.2. Mouse Dosing

Female Swiss Webster (SW) mice aged 6 weeks were purchased from Jackson Laboratories (Sacramento, CA, USA). Mice were acclimatized for two weeks prior to the study and were housed in a climate-controlled room with 12 h light-dark cycle with access to food and water *ad libitum*. All animal experiments were performed in AAALAC-accredited facilities and under the University of New Mexico Institutional Animal Care and Use Committee approved protocol (Protocol # 17-200555-HSC).

Mice were randomly assigned to three different groups to be imaged at three different time points (1, 4, and 24 h) after pulmonary administration with *n* = 4 per group per time point: The groups consisted of: (1) oropharyngeal aspiration, and IT instillation using (2) the MicroSprayer^®^ Aerosolizer (Penn-Century Inc., Wyndmoor, PA, USA), and (3) the BioLite Intubation System (Braintree Scientific, Braintree, MA, USA). Additionally, longitudinal three-dimensional imaging was performed on a single mouse from each group at 1, 4, and 24 h. Three mice were sacrificed at each time point after administration of fluorescent microparticles. Lung, spleen, kidney, and liver tissues were collected and imaged using the IVIS Spectrum in vivo imaging system (PerkinElmer, Waltham, MA, USA). 

**Figure 1:**
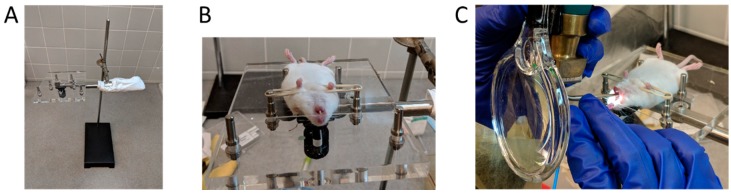
Steps for mouse intubation. (**A**) Set-up of the intubation platform; (**B**) Anesthetized mouse placed on its back and secured on the intubation platform; and (**C**) Visualization of the trachea using a small animal laryngoscope, with an attached 3X magnifying glass, to facilitate administration of the test agent.

For all the methods, mice were anesthetized with ketamine/xylazine (90–100 and 9–10 mg/kg respectively) prior to pulmonary dosing. Anesthetized mice were placed on their back and secured on the intubation platform (Penn-Century Inc., Wyndmoor, PA, USA) with a rubber band (Figure 1A,B). The tongue was rolled out of the mouth with a Q-tip and the tracheal opening was visualized by inserting a small animal laryngoscope (Model LS2; Penn-Century Inc., Wyndmoor, PA, USA) with an attached 3X magnifying glass [25,26] (Figure 1C).

#### 2.2.1. MicroSprayer^®^ Aerosolizer

**Figure 2:**
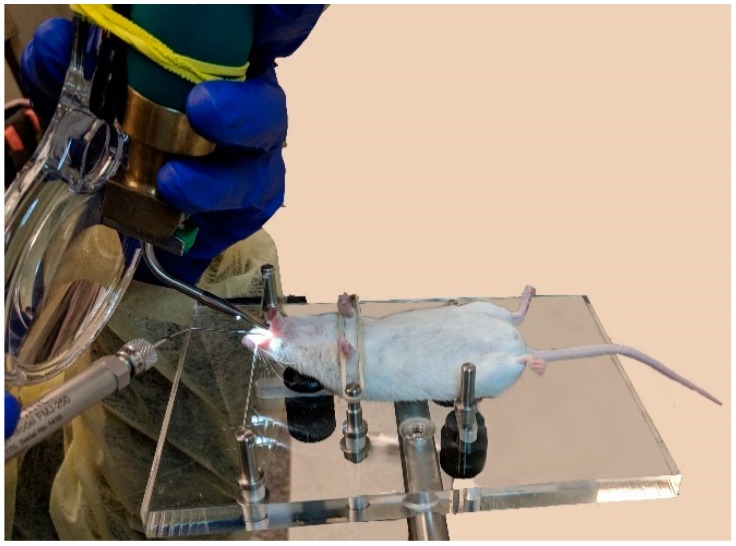
Mouse intubation using the MicroSprayer^®^ Aerosolizer (Penn-Century, Wyndmoor, PA, USA). The microsprayer is inserted into the trachea of the mouse with the help of a small animal laryngoscope.

The microsprayer was assembled according to the manufacturer’s instruction and as performed previously [7]. Once the tracheal opening was visualized using the laryngoscope, the microsprayer delivery tube was inserted gently into the trachea of the mouse, proximal to the carina, until the curve of the delivery tube was positioned at the incisors. Microspheres (50 μL) were aerosolized into the lungs by depressing the microsprayer plunger with a constant force (while another person was holding the syringe steady so as not to injure the mouse trachea), and waiting 5 s after delivery before removing the microsprayer from the mouse trachea (Figure 2).

#### 2.2.2. BioLite Intubation System

**Figure 3:**
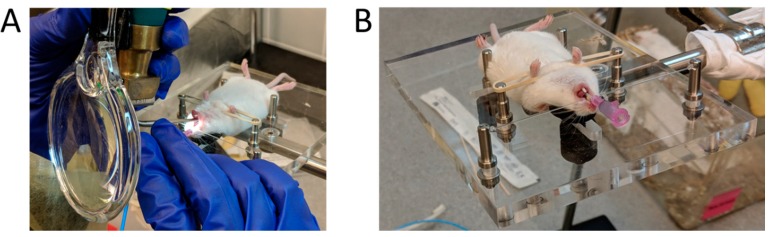
Mouse intubation using the BioLite Intubation System (Braintree Scientific Ltd., Braintree, MA, USA). (**A**) Visualization of the trachea using a small animal laryngoscope and a fiber-optic stylet. (**B**) Cannula inserted into the trachea of the mouse, ready for attaching the syringe and administration of the test agent.

The BioLite Intubation System comprises of a fiber optic illuminator, fiber optic stylet, and an intratracheal catheter tube. The catheter tube is place over the fiber optic stylet/guide wire which provides lighting of the oropharynx facilitating intubation and drug administration into the lungs [27]. The intubation system was assembled according to the manufacturer’s instruction and 50 μL of fluorescent microspheres were loaded into a syringe before each intubation. Once the tracheal opening was visualized using the laryngoscope (Figure 3A), the BioLite fiber-optic stylet with the attached intubation catheter was inserted gently into the trachea of the animal, proximal to the carina, until the end of the catheter tube was positioned at the incisors. The fiber-optic stylet was removed, leaving the intubation catheter in the trachea (Figure 3B). The loaded syringe was then attached to the catheter and the microspheres were delivered into the lungs by compressing the plunger with constant force (while another person held the syringe steady so as not to injure the mouse trachea), and waiting 5 s after delivery before removing the intubation delivery tube from the trachea.

#### 2.2.3. Oropharyngeal Aspiration

**Figure 4:**
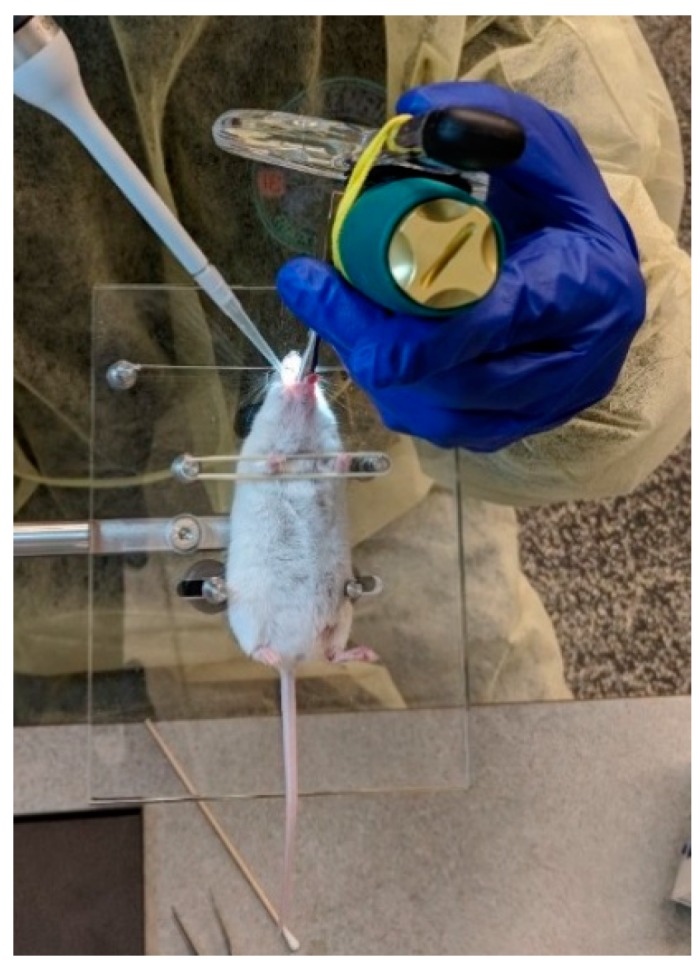
Administration of fluorescent microparticles by the ‘oropharyngeal aspiration’ method. The tracheal opening was visualized with the help of a small animal laryngoscope.

A pipette tip (200 μL) was loaded with 50 μL of fluorescent microspheres before each aspiration. The anesthetized mice was placed on the intubation platform and the tongue is rolled to one side using a Q-tip. Once the tracheal opening was visualized using the laryngoscope, the pipette tip was placed at the tracheal opening (Figure 4) and the microspheres were delivered into the trachea for lung deposition. During the deposition, the nasal passage was occluded by a fingertip (by another person), forcing the mice to breathe through the mouth thus enabling the deposition of microparticles into the lungs. The fingertip was released after one breath had been completed [21].

### 2.3. Fluorescence Imaging

Mice administered with the near-IR fluorescent microspheres were imaged using an IVIS Spectrum small-animal imaging system. Excitation (λ_ex_) of 745 nm and emission (λ_em_) of 820 nm filters were used. Epi-illumination settings used for image acquisition were exposure time (0.5–20 s), binning factor, f-stop (2), and field of view (22.8 cm). For three-dimensional (3D) image acquisition, fluorescent and photographic images were acquired and overlaid. Data analysis was performed using the Living Image 4.0 software. The pseudo-colored images represent the spatial distribution of photon counts.

### 2.4. Statistical Analysis

The three methods of pulmonary administration—oropharyngeal aspiration, the MicroSprayer^®^ Aerosolizer, and the BioLite Intubation System—were compared at three time points (1, 4, and 24 h). The mean of total flux (the measure of fluorescence, at least three mice per time point per method) between each method was compared for significant differences using one-way ANOVA with Tukey’s multiple comparison test.

## 3. Results

### 3.1. Whole Animal Imaging

**Figure 5:**
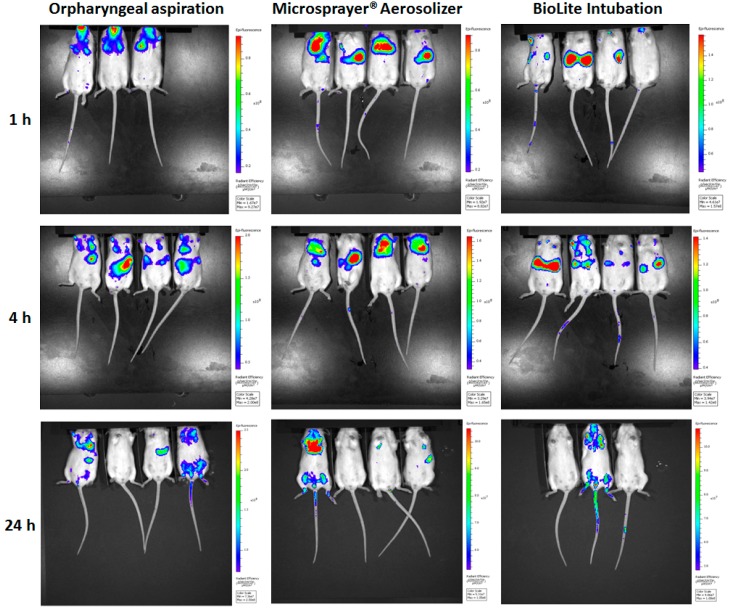
Whole animal images of mice acquired using IVIS Spectrum at 1, 4, and 24 h after pulmonary administration of near-infrared fluorescent Degradex^®^ poly(d,l-lactide-co-glycolide) (PLGA) microspheres using oropharyngeal aspiration, the MicroSprayer^®^ Aerosolizer, and the BioLite Intubation System.

Animals were administered fluorescent microparticles and were imaged using the IVIS Spectrum imaging system while under anesthesia. As seen in Figure 5, all three methodologies showed fluorescence in the lung fields at the 1 and 4 h time points. Importantly, mice that were administered microparticles via the oropharyngeal aspiration showed deposition in the oral cavity, in addition to the trachea and the lungs. However, we did not observe particle deposition in the oropharyngeal region of mice that were administered microparticles via IT instillation (MicroSprayer^®^ Aerosolizer and BioLite Intubation System).

**Figure 6:**
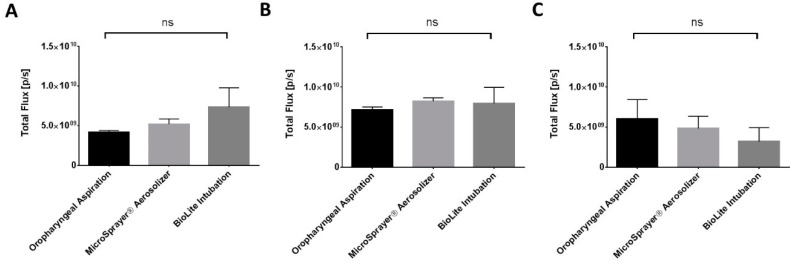
Total fluorescence flux (ρ/s) (oral cavity, trachea, and the lungs) in mice at (**A**) 1 h, (**B**) 4 h, and (**C**) 24 h after pulmonary administration of near-infrared fluorescent Degradex^®^ poly(d,l-lactide-co-glycolide) (PLGA) microspheres using oropharyngeal aspiration, the MicroSprayer^®^ Aerosolizer, and the BioLite Intubation System (*n* = 3–4, mean ± SEM, one-way ANOVA, Tukey’s multiple comparison test).

The total fluorescence flux was quantified by drawing a region of interest (ROI) that included the oral cavity (oropharyngeal region), trachea, and the lungs. The total flux (ρ/s) at 1 h after pulmonary administration was 4.18 × 10^9^ ± 2.08 × 10^8^ for oropharyngeal aspiration, 5.21 × 10^9^ ± 6.38 × 10^8^ for the MicroSprayer^®^ Aerosolizer, and 7.33 × 10^9^ ± 2.45 × 10^9^ for BioLite intubation. At the 4 h and 24 h time points, the total flux (ρ/s) was 7.17 × 10^9^ ± 3.38 × 10^8^ (4 h) and 6.07 × 10^9^ ± 2.37 × 10^9^ (24 h) for oropharyngeal aspiration, 8.24 × 10^9^ ± 4.22 × 10^8^ (4 h) and 4.85 × 10^9^ ± 1.50 × 10^9^ (24 h) for the MicroSprayer^®^ Aerosolizer, and 7.97 × 10^9^ ± 2.00 × 10^9^ (4 h) and 3.21 × 10^9^ ± 1.72 × 10^9^ (24 h) for BioLite intubation. The total flux was similar between all the delivery methods and at all time points (non-significant, one-way ANOVA) (Figure 6).

### 3.2. Lung Imaging

**Figure 7:**
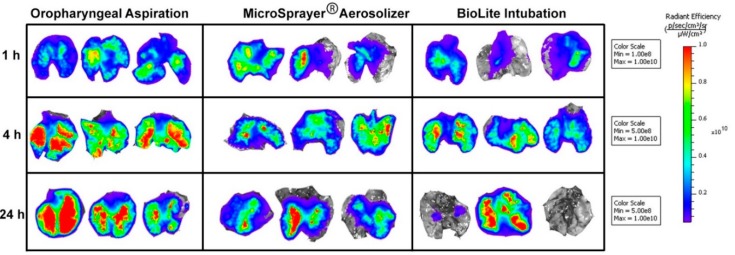
Images of excised lungs of mice acquired using IVIS Spectrum at 1, 4, and 24 h after pulmonary administration of near-infrared fluorescent Degradex^®^ poly(d,l-lactide-co-glycolide) (PLGA) microspheres using oropharyngeal aspiration, the MicroSprayer^®^ Aerosolizer, and the BioLite Intubation System.

At 1, 4, and 24 h after pulmonary administration of the fluorescent microparticles, mice were sacrificed and the lung tissue was excised and imaged using IVIS Spectrum (Figure 7). In addition, at 4 and 24 h time points, liver, spleen, and kidneys were excised and imaged to quantify microparticle distribution. 

**Figure 8:**
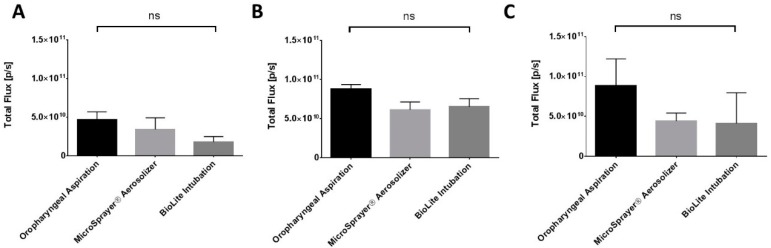
Total flux (ρ/s) of fluorescence in excised lungs of mice at (**A**) 1 h, (**B**) 4 h, and (**C**) 24 h after administration of near-infrared fluorescent Degradex^®^ poly(d,l-lactide-co-glycolide) (PLGA) microspheres using oropharyngeal aspiration, the MicroSprayer^®^ Aerosolizer, and the BioLite Intubation System (*n* = 3–4, mean ± SEM, one-way ANOVA, Tukey’s multiple comparison test).

The total fluorescence flux was quantified by drawing a region of interest (ROI) around the entire excised tissue. The total flux (ρ/s) at 1 h after pulmonary administration was 4.64 × 10^10^ ± 1.03 × 10^10^ for oropharyngeal aspiration, 3.41 × 10^10^ ± 1.49 × 10^10^ for the MicroSprayer^®^ Aerosolizer, and 1.77 × 10^10^ ± 7.10 × 10^9^ for BioLite intubation. At the 4 and 24-h time-points, the total flux (ρ/s) was 8.74 × 10^10^ ± 6.00 × 10^9^ (4 h) and 8.82 × 10^10^ ± 3.37 × 10^10^ (24 h) for oropharyngeal aspiration, 6.10 × 10^10^ ± 1.01 × 10^10^ (4 h) and 4.40 × 10^10^ ± 1.01 × 10^10^ (24 h) for the MicroSprayer^®^ Aerosolizer, and 6.50 × 10^10^ ± 1.03 × 10^10^ (4 h) and 4.12 × 10^10^ ± 3.83 × 10^10^ (24 h) for BioLite intubation. The total flux was similar between all the delivery methods and at all time points (non-significant, one-way ANOVA) (Figure 8). We observed a trend towards higher deposition in the excised lung with the oropharyngeal aspiration method at all time points; however, the differences were not significant. Moreover, the total flux at the 4 and 24 h time points in the liver, spleen, and kidney was very minimal and not significant between the three different methods of administration [28].

### 3.3. Three-Dimensional Longitudinal Imaging

**Figure 9:**
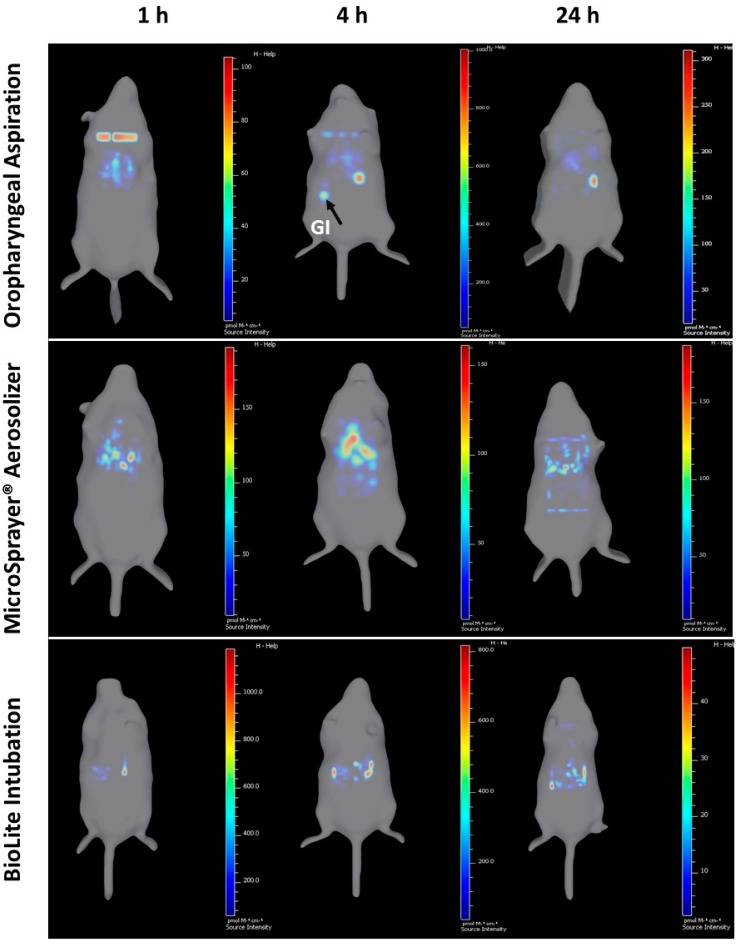
Three-dimensional (3D) longitudinal imaging of mouse at 1, 4, and 24 h using IVIS Spectrum after administration of near-infrared fluorescent Degradex^®^ poly(d,l-lactide-co-glycolide) (PLGA) microspheres using oropharyngeal aspiration, the MicroSprayer^®^ Aerosolizer, and the BioLite Intubation System (3D video available as Supplementary Video S1). Gastrointestinal (GI) deposition using the oropharyngeal aspiration technique is shown with an arrow.

One mouse from each of the pulmonary delivery method was analyzed longitudinally for 24 h using 3D imaging with the IVIS Spectrum. As shown in Figure 9, at 1 h post-administration, the mouse that received the microparticles via the oropharyngeal aspiration had lung deposition as well as deposition in the oropharyngeal region, which over time (4 h) is seen in the gastrointestinal (GI) tract (Figure 9: GI deposition shown by an arrow). Further, the amount of fluorescence in the lungs decreased over time, possibly due to clearance from the lung. For the MicroSprayer^®^ Aerosolizer, we did not observe any microparticle deposition in the oral cavity. The majority of the dose, as it was administered close to the bifurcation of the trachea, was visible in the trachea and the two lobes of the lungs. The accumulation of the microparticles in the lung was more visible at the 4 h time point after administration; whereas after 24 h, similar to the oropharyngeal aspiration group, the microparticles were partially cleared from the lung tissue. Interestingly, mice that were administered microparticles using BioLite intubation showed a small amount of particles in the deeper regions of the lungs with minimal fluorescence detected in the oral cavity, trachea, or the upper respiratory tract possibly due to the low dose delivered from the device. This highlights the challenge faced by researchers in the pulmonary delivery preclinical field, and the expertise and training required to use IT instillation devices.

## 4. Discussion

The first human inhaler in the form of a pressurized metered dose inhaler (pMDI) (Riker Laboratories, Inc., Northridge, CA, USA, now 3 M Drug Delivery Systems) was introduced in 1956 and heralded the beginning of the modern pharmaceutical aerosol industry [29]. Since the introduction of the first inhaler prototype, the aerosol industry has experienced dramatic growth, with the subsequent introduction of nebulizers and dry powder inhalers. In recent years, the lung has been used as a target organ to deliver vaccines and immunotherapeutics because it is the primary port of entry for many infectious pathogens [1].

Microparticle-based vaccines and therapeutics offer several advantages over their traditional counterparts. Specifically, microparticle-based vaccines have shown better immunogenicity due to their comparable size to many pathogens, making them excellent stimulators of the human immune system. Further, microparticles can be optimized to release the antigen slowly in vivo, resulting in persistent triggering of the immune system [8]. Despite the interest in evaluating particulate-based vaccines for pulmonary delivery, successful translation of drug and vaccine candidates from the preclinical to the clinical trial stage has been abysmal. One of the reasons for the poor clinical translation is the lack of effective pulmonary delivery tools for preclinical studies. Further, a lack of standardized and effective inhalation devices for preclinical use has resulted in poor reproducibility between laboratories and is a huge impediment in the successful translation of pulmonary formulations to humans [20]. Here, we successfully evaluated three commonly used preclinical pulmonary devices/techniques in mice with respect to respiratory tract deposition and distribution patterns using IVIS imaging.

As mentioned earlier, preclinical pulmonary delivery devices employ either passive or direct inhalation techniques. Passive inhalation allows the animal to breathe the test agent normally over a period, whereas direct inhalation forces the test agent into the upper respiratory tract of the animal using a cannula. With passive inhalation techniques, significant drug losses occur in the reservoir, tubing of the aerosol generator, delivery accessories, and the nasopharyngeal region of the animal, resulting in poor delivery efficiency and variable control over the dose delivered. These drawbacks limit the applicability of passive inhalation techniques for evaluating drug candidates with a narrow therapeutic window. This is because passive inhalation techniques lead to significant variations in the delivered dose and, further, this dose cannot be determined precisely. This prohibits the use of passive inhalation devices for testing novel vaccines and immunotherapies in preclinical models since it requires precise dose titration. Moreover, with these devices, there is extra-pulmonary exposure to other mucosal tissues, such as the oropharynx, which may result in the generation of a non-specific immune response [22]. 

Direct inhalation techniques allow for precise quantification of the dose delivered to the lungs as the test agent is administered directly into the upper respiratory tract, thus bypassing the nasopharynx [30]. Direct inhalation techniques in preclinical models include IT instillation and tracheostomy. As mentioned earlier, these techniques require technical expertise and anesthetization of animals prior to administration, and repeated dosing may cause inflammation and injury to the trachea. An alternative technique to IT installation and tracheostomy is oropharyngeal aspiration, which can potentially overcome some of the challenges mentioned above [23,24,31]. Lakatos et al. compared oropharyngeal aspiration to IT instillation to establish a silica-induced fibrosis mouse model and observed that administration of silica particles by aspiration resulted in a more uniform pulmonary distribution with minimal intra-animal variability [32]. In contrast, Robbe et al. found that IT instillation using a microsprayer resulted in a more homogeneous bleomycin-induced lung fibrosis mouse model with higher-grade damage when comparing aspiration to IT instillation [33]. Vartiainen et al. used aspiration to administer silicon dioxide to induce pulmonary fibrosis, after which they used aspiration to deliver tilorone as a therapy to treat fibrosis. The authors observed a significant reduction in the histological fibrosis score with the aspiration delivery method [34]. Further, Chakravarthy et al. have used the aspiration technique to deliver formulations to the lungs and target the alveolar macrophages [35]. All these preclinical studies demonstrate the use of preclinical pulmonary delivery devices to be arbitrary, with variable results achieved [20].

In our study, we observed the deposition of the microparticles in the oral cavity after oropharyngeal aspiration. The dose delivered to the oral cavity was ultimately swallowed and reached the GI tract at the 4 h time point (Figure 9). Although aspiration is a less invasive technique than IT instillation, one of the drawbacks with such a method is the inadvertent delivery of the drug to the oral cavity. Further, it is difficult to precisely estimate the distribution of the dose between the GI tract, the trachea, and the lungs. An accurate estimation of the dose delivered to the lungs is crucial to determine the on-target/local effects, especially when evaluating vaccines and immunotherapies. Therefore, despite the advantages of aspiration method over IT instillation, our study suggests that the lack of precise quantification of the delivered dose in the lungs limits the applicability of this method.

IT instillation methods for drug and vaccine delivery to the lungs have been widely reported in the literature [3,7,36,37,38,39]. The most commonly used devices are the Penn-Century MicroSprayer^®^ Aerosolizer and the BioLite Intubation System; however, the MicroSprayer^®^ device was discontinued in 2016 and is currently not available for purchase. We have previously used this device to deliver drugs and vaccines in small animals including mice and guinea pigs [7,18]. With the BioLite intubation system, the dose is administered using a syringe that is attached to a cannula positioned in the trachea. When depressing the plunger of the syringe, the suspension or solution drips from the cannula into the trachea; this can sometimes lead to drug being deposited in only one of the lung lobes. However, the microsprayer aerosolizes the suspension or solution while being placed in the trachea, thus increasing the possibility of the dose entering both lung lobes. Rajapaksa et al. delivered plasmid DNA vaccine using the BioLite Intubation System into rat lungs and achieved effective systemic and mucosal antibody titers [40]. However, it is not known if uniform distribution of the vaccine in both the lung lobes is required to generate an effective systemic and local immune response. In another study, Sadhuka et al. compared the BioLite Intubation System and a passive inhalation exposure system (nose-only exposure) to test the efficacy of epidermal growth factor receptor (EGFR)-targeted superparamagnetic iron oxide nanoparticles in a lung cancer animal model. Passive inhalation exposure was able to achieve uniform distribution of the nanoparticles throughout the lungs compared to the BioLite Intubation System, where the majority of the dose was deposited in the upper airways with limited deposition in the peripheral airways. Despite the limited peripheral lung distribution of the agent with the BioLite Intubation System, the authors observed that the IT instillation method had a 5-fold higher deposition compared to the passive inhalation exposure [41]. Since small rodents (including mice and rats) are obligatory nose breathers [20], inhalation exposure (via the nasal passage) could potentially result in low drug deposition in the lungs. Therefore, IT instillation techniques are the preferred method to achieve higher and precise dosing of the agent into the lungs. However, as mentioned earlier, it is important to note that IT instillation techniques in preclinical models are markedly different from inhalation methodologies used in humans. Table 1 lists the similarities and differences with regards to ease of administration and respiratory tract deposition and distribution pattern between the three methods evaluated.

The IVIS Spectrum animal imager used in our studies has a few limitations. This imaging modality is known to be semi-quantitative since the fluorescence quantified depends on the excitation and emission wavelength of the dye, and the depth of the tissue in which the particles are being analyzed. Like other imaging modalities, this imager has limitations with regards to sensitivity, spatial resolution, and the ability to accurately quantify the agent in vivo. Future studies should, therefore, include quantification of the in vivo dose deposited in various organs, including the oral cavity, trachea, lung lobes, stomach, kidneys, spleen, and liver to confirm the data obtained here using IVIS Spectrum for the three delivery methods. This can be achieved by using analytical techniques such as HPLC and LCMS; such a quantification would be complementary to the imaging techniques that allow for the precise spatial distribution of the particles in real time in vivo.

## Figures and Tables

**Table 1:** Similarities and differences between oropharyngeal aspiration, the MicroSprayer^®^ Aerosolizer, and the BioLite Intubation System.

	Oropharyngeal Aspiration	MicroSprayer^®^ Aerosolizer	BioLite Intubation System
**Ease of Administration**	Easy to use with minimal expertise; only requires a pipette (a small animal laryngoscope facilitates visualization of the oropharynx/trachea)	Technical expertise needed; requires a small animal laryngoscope, however, the device is currently discontinued	Technical expertise required; requires a small animal laryngoscope and the purchase of a BioLite Intubation System
	Drug suspension/solution is placed at the back of the oropharynx; mice are forced to breathe by occluding nose with a fingertip, facilitating drug delivery into the lungs	The delivery tube is inserted gently into the trachea and the drug suspension/solution is forced into the lungs	The intubation catheter is gently inserted into the trachea with the help of a fiber-optic stylet/guide wire. The stylet is slowly removed and a drug suspension/solution loaded syringe is attached to the catheter and delivered by compressing the syringe plunger
**Respiratory Tract Deposition and Distribution**	Possible deposition in the oral cavity, in addition to trachea, and the lungs	Showed deposition in the trachea and the lungs (no deposition in the oral cavity as the delivery tube is inserted into the trachea)	Showed deposition in the trachea and the lungs (no deposition in the oral cavity as the intubation catheter is inserted into the trachea)
	3D imaging shows microparticles reaching the GI tract at 4 and 24 h, indicating GI deposition along with tracheal and lung deposition	3D imaging shows majority of the microparticle deposition in trachea and the lungs	3D imaging shows majority of the microparticle deposition in trachea and the lungs
	No significant differences in total flux/deposition of microparticles at 1, 4, and 24 h in whole animal and excised lungs, liver, spleen, and kidneys		

## Supplementary Materials

The following are available online at http://www.mdpi.com/2076-393X/6/3/41/s1, Video S1: Three-dimensional (3D) video of fluorescent microspheres deposition in mouse at 1, 4, and 24 h using IVIS Spectrum using oropharyngeal aspiration, the MicroSprayer^®^ Aerosolizer, and the BioLite Intubation System.
